# The Mechanosensory Subgenual Organ Complex in the Stick Insect *Bacillus rossius* (Phasmatodea): Neuroanatomy and Functional Morphology

**DOI:** 10.1002/cne.70126

**Published:** 2026-01-10

**Authors:** Johannes Strauß, Peter T. Rühr

**Affiliations:** ^1^ AG Integrative Sensory Physiology, Institute for Animal Physiology Justus Liebig University Gießen Gießen Germany; ^2^ Center for Mind, Brain and Behavior (CMBB) University of Marburg and Justus Liebig University Gießen Gießen Germany; ^3^ Bonn Institute for Organismic Biology BIOB, Section II: Animal Biodiversity University of Bonn Bonn Germany

**Keywords:** biomechanics, biotremology, chordotonal organ, SCR_002798, SCR_003070, SCR_014235, SCR_014567, SCR_017999, sensory evolution, vibration

## Abstract

The subgenual organ complex is an elaborate mechanosensory complex in the insect leg containing chordotonal organs. In stick and leaf insects (Phasmatodea), it includes the subgenual organ and the distal organ. This study documents the neuroanatomy and functional morphology of the subgenual organ complex in the stick insect *Bacillus rossius* (Bacillidae: Bacillinae) by axonal tracing and micro‐computed tomography. It also considers the first report on the subgenual organ complex in stick insects that reported a relatively simple organization of the sensory organ and its nerves on the basis of histological sections. Our findings show the neuroanatomy and nerve pattern of the subgenual organ complex in *B. rossius* with a subgenual organ and a distal organ. The subgenual organ is placed in the hemolymph channel. The distal organ is also located in the hemolymph channel, and it has several attachment elements, linking it to the cuticle of the tibia, the tibial tracheae, and the subgenual organ. The connections to the tibia may form an input pathway for vibrations transmitted over the cuticle, whereas the position in the hemolymph channel and the connection to the subgenual organ indicate a mechanical activation by vibrations transmitted via the hemolymph. Overall, the axonal tracing preparations document neuroanatomical details for *B. rossius* and resolve the numbers of sensilla in the sensory organs, the length of the distal organ, and confirm a single nerve branch for the subgenual organ. The data provide support for a consistent organization of the subgenual organ complex within stick insects.

## Graphical abstract

The subgenual organ complex in orthopteroid insects consists of few mechanosensory organs. In the stick insect *Bacillus rossius*, the subgenual organ and distal organ include ∼70 sensilla, and both organs are located in the hemolymph channel with several connections to the leg cuticle. The neuroanatomy and functional morphology of the sensory organs resemble that of other stick insects and support a shared vibrosensory function.

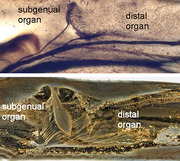

## 1 Introduction

Stick insects or Phasmatodea are important study species for neurophysiology, locomotion, and its sensory regulation (Ayali et al. 2015; Dürr et al. 2019; Büschges and Ache 2025). Few species are established as model species, including the Indian stick insect *Carausius morosus* Brunner von Wattenwyl, 1907. *Bacillus rossius* Rossi, 1790, is also an important experimental model (Carlberg 1986a; Bradler and Buckley 2018), in particular, for studying the evolution of insect parthenogenetic reproduction and speciation (Scali 1982; Carlberg 1986b, 1987; Mantovani et al. 2001; Scali et al. 2003; Andersen et al. 2006; Forni et al. 2022; Lavanchy et al. 2024). In addition, Phasmatodea are an important taxon for the analysis of evolutionary diversification and convergence (Whiting et al. 2003; Boisseau et al. 2025). Although morphological convergence was problematic for the understanding of the Phasmatodea phylogeny (e.g., Bradler and Buckley 2018), molecular phylogenetics have supported major clades relating to biogeography (New World stick insects: Occidophasmata and Old World stick insects: Oriophasmata) (Simon et al. 2019; Tihelka et al. 2020; Boisseau et al. 2025).

Stick insects are analyzed for the leg mechanoreceptors involved in the control of locomotion and vibration detection, including the chordotonal organs that are internal sensory organs. They consist of scolopidial sensilla in variable numbers (Strauß, Stritih‐Peljhan, et al. 2021; Goulding et al. 2025). In stick insects and related groups of Polyneoptera, the subgenual organ complex occurs in the proximal tibia with the chordotonal subgenual organ and several additional organs. The subgenual organ is an important vibrosensory organ in most insects (Shaw 1994a; Eberhard et al. 2010; Strauß, Stritih‐Peljhan, et al. 2021; Virant‐Doberlet et al. 2023), whereas it can also respond to airborne sound of relatively low frequencies (Shaw 1994b; Kalmring et al. 1994; Stumpner 1996). The additional chordotonal organs are vibrosensitive (Schnorbus 1971) or bimodal (Kalmring et al. 1994). In stick insects, the subgenual organ complex consists of two chordotonal organs with an elaborate neuroanatomy: The subgenual organ forms a hemi‐circle of sensilla in the tibia, and the distal organ is placed distally to the subgenual organ with linear sensilla along the main axis of the tibia (Strauß and Lakes‐Harlan 2013; Strauß, Moritz, et al. 2021). Among Neophasmatodea (Old World and New World stick insects), the neuroanatomy of these sensory organs is very similar (Strauß 2023a). *B. rossius* (Bacillidae: Bacillinae) is also relevant for comparative studies of the sensory system as it was the first stick insect species investigated for the subgenual organ complex, mainly based on histological sections (Oyen 1901). This first study differed notably in the interpretation of the subgenual organ neuroanatomy by ascribing all sensilla to the subgenual organ (“subgenual organ I and II”), and the distal organ was identified later in *C. morosus* on the basis of homology to sensory organs in other Polyneoptera (Friedrich 1929). Oyen (1901) described the different orientations of sensilla in the tibia (subgenual organ: transversal, distal organ: longitudinal) as well as the linear arrangement of distal sensilla. However, the study also included some neuroanatomical details contrasting with most or all other stick insects investigated so far: The origin of the nerve for the subgenual organ complex from the main leg nerve was identified more proximally in the femur; two short nerve branches from this nerve are associated with the sensilla in the organ complex; the distal organ (“subgenual organ II”) is rather short with a length similar to the subgenual organ; and the sensory organ is placed on the leg tracheae. On the basis of data from *C. morosus*, Friedrich (1929) later noted some differences to *B. rossius*: The sensory neurons of the “subgenual organ II” were identified as the distal organ, and a second subgenual organ nerve branch was suggested to be present in *B. rossius* as well (Friedrich 1929). However, the nerve pattern in Phasmatodea can include either one or two subgenual nerve branches (Strauß 2025). These traits for the nerve pattern and organ morphology can be clearly identified in the sensory organs by axonal tracing. The distal organ prominently extends distally from the subgenual organ, whereas the numbers of sensilla vary between species from 16 (Strauß and Lakes‐Harlan 2013) to ∼30 (Strauß 2025). The short set of sensilla depicted by Oyen (1901) for the distal organ is unusual compared to other Phasmatodea and would indicate a low number of sensilla in the distal organ. To study, the subgenual organ complex in *B. rossius* allows us to specify the neuroanatomy for this species, document the nerve pattern, and quantify the number of sensilla. With our new evidence on such neuroanatomical features, we address the diversity of the sensory complex in Phasmatodea.

Such data add to the taxon sampling on sensory organs for stick insects in addition to the model species established in neurobiology research, such as *C. morosus* (Lonchodidae: Lonchodinae) and *Sipyloidea chlorotica* Audinet‐Serville, 1838 (Lonchodidae: Necrosciinae). The phylogenetic position of *Bacillus* differs between phylogenetic studies within Oriophasmata in clades as sister lineage to Phylliinae (Simon et al. 2019) or the African/Malagasy clade (Boisseau et al. 2025), or to Pachymorphinae (Robertson et al. 2018) or Diapheromerinae (Büscher et al. 2018). Data on differences in the nerve pattern, organ size, or sensilla numbers may be used with future data from these groups to support taxonomic affinities. We here study the subgenual organ complex by axonal tracing and micro‐computed tomography to document the neuroanatomy and functional morphology of the sensory organs in *B. rossius*, with specific focus on features interesting for comparative and evolutionary neurobiology. These concern (i) the overall organization of the subgenual organ complex, (ii) the origin of the nerve for the sensory organs, (iii) the nerve pattern of the subgenual organ, (iv) the organization and size of the distal organ, and (v) the attachment and connection of the distal organ to cuticle and the leg trachea.

## 2 Materials and Methods

### 2.1 Stick Insects

**FIGURE 1:**
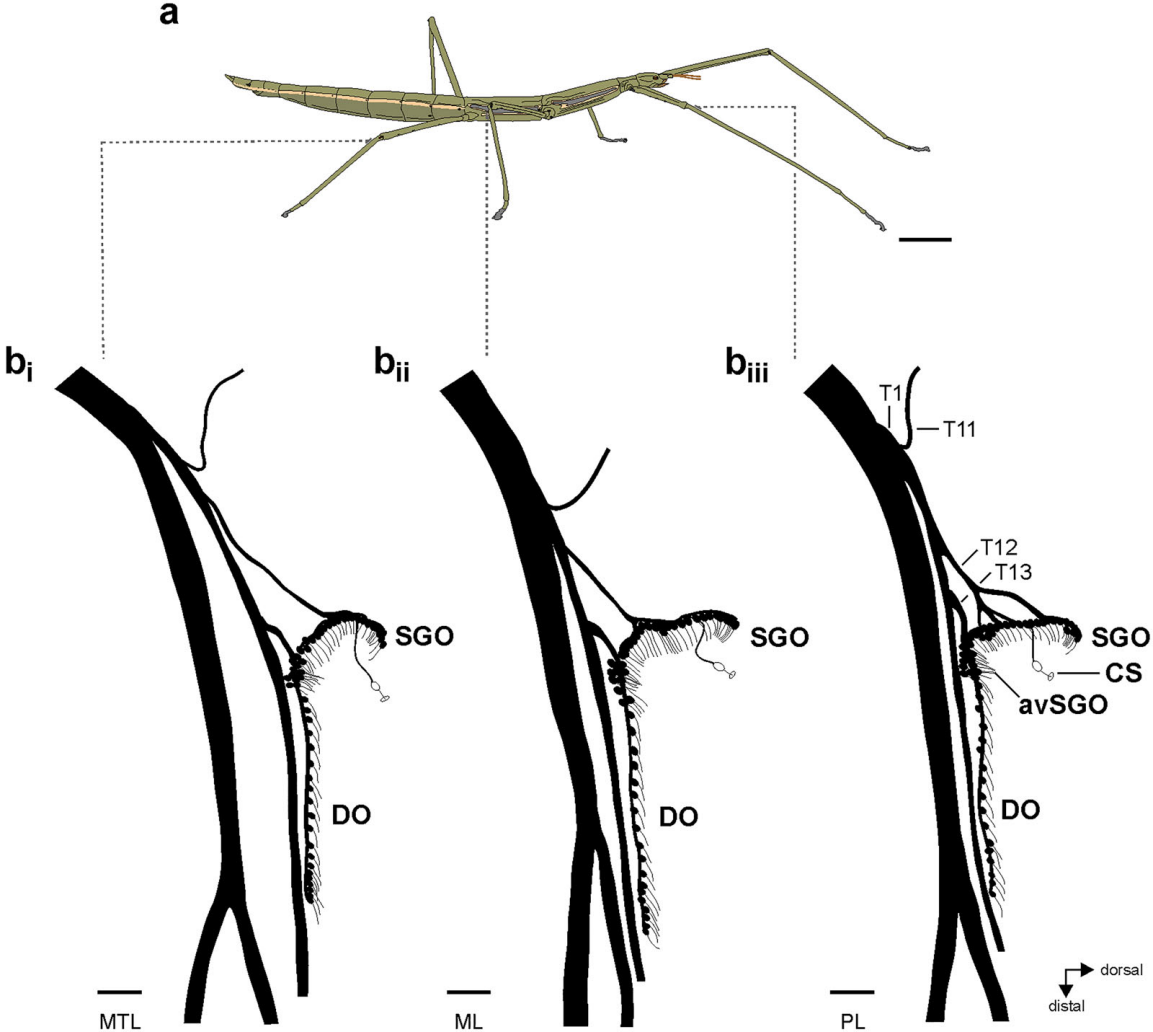
The subgenual organ complex in *Bacillus rossius*. (a) Habitus of a female *B. rossius*. (b) Schematic of the subgenual organ complex in the (b_i_) mesothoracic leg/hindleg, (b_ii_) mesothoracic leg/midleg, and (b_iii_) prothoracic leg/foreleg. Nerve branches and their sub‐branches from nervus cruris are identified by successive numbering (T1, T11, T12, T13) for the prothoracic leg (b_iii_). Scales: (a) = 1 cm; (b) = 100 µm. avSGO, anterior‐ventral subgenual organ; CS, campaniform sensillum; DO, distal organ; ML, mesothoracic leg; MTL, metathoracic leg; PL, prothoracic leg; SGO, subgenual organ.

This study investigated the neuroanatomy of adult female *B. rossius* (Figure 1a). Insects were raised in terraria at the Institute for Animal Physiology, Justus Liebig University Gießen, Gießen, Germany, at room temperatures between 21°C and 23°C. They were fed with leaves of bramble ad libitum and sprayed daily with water.

The experiments in the present study comply with the principles of animal care of the Justus Liebig University Gießen, Germany, as well as with the current law of the Federal Republic of Germany.

### 2.2 Neuroanatomy and Axonal Tracing

Sensory organs were stained by retrograde axonal tracing with cobalt solution (Pitman et al. 1973; Altman and Tyrer 1980). Overall, 5% cobalt solution (cobalt(II) chloride hexahydrate, from Merck, Darmstadt, Germany) in Aqua dest was applied to the cut nerve (nervus cruris, the major leg nerve). The tracing follows the procedure described previously for legs of stick insects (Strauß 2020). For axonal tracing, the legs were cut off at the coxa‐trochanter joint with scissors, following brief cold‐anesthesia at 4°C for 5 min.

Preparation and tracing were carried out on isolated legs fixed with insect pins in a glass dish filled with Sylgard (Sylgard 184, Suter Kunststoffe AG, Fraubrunnen, Switzerland). Prior to dissection, the legs were covered with *Carausius* saline (pH = 7.4; Bässler 1977). The ventral cuticle of the femur was cut with a piece of a blade (Feather FA‐10, 0.1 mm, Feather, Osaka, Japan), and the tendon and flexor muscles (Bässler 1977) were removed with forceps (Dumont #5, Fine Science Tools, Heidelberg, Germany). The nervus cruris runs in the middle of the femur in stick insects (Bässler 1977) and can, therefore, be accessed from the ventral side of the femur. The nervus cruris was cut above the femur‐tibia joint, and the cut end was transferred into a glass capillary filled with cobalt solution (see above). Preparations were incubated in a moist chamber at 4°C for 48 h. Cobalt was then precipitated by a 1% ammonium sulfide solution (Alpha Aesar, Karlsruhe, Germany) (in *Carausius* saline) with incubation for 15 min. The legs were rinsed twice in phosphate buffer (0.04 mol/L Na_2_HPO_4_, 0.00574 mol/L NaH_2_PO_4_·2 H_2_O, Merck, Darmstadt, Germany; pH = 7.4) and fixed in chilled paraformaldehyde (4%, Sigma Aldrich, St. Louis MO, USA) for 1 h. In preparation for microscopy, the legs were dehydrated in a graded series of ethanol (Carl Roth, Karlsruhe, Germany) and cleared methyl salicylate (Merck, Darmstadt, Germany).

In total, legs from 10 insects were used in tracing experiments for 51 leg preparations (Table 1). For counts of sensilla, preparations from nine stick insects were analyzed with one of the legs from each thorax segment, and preparations from one insect were excluded in which the prothoracic legs were insufficiently stained to count the individual sensilla. The analysis of the nerve pattern excludes two preparations from metathoracic legs in which the nerve branches could not be clearly detected.

**TABLE 1:** Summary of leg preparations analyzed for neuroanatomical aspects of the sensory organs in the subgenual organ complex.

	Prothoracic leg	Mesothoracic leg	Metathoracic leg	Legs total
***n* (Insects)**	9	10	10	
***N* (legs stained)**	15	17	19	51
***n* (Legs selected for sensilla count)**	9	9	9	27
***n* (Legs selected for nerve pattern)**	15	17	17	49

The nomenclature of nerve branches from the nervus cruris in the tibia uses the consecutive numbering of nerve branches in the proximal segments of the leg (Bässler 1983) as used previously (Strauß 2020; Strauß, Moritz, et al. 2021) (T1, T2). The terminology of the section levels of the tibia is used as indicated by Ball and Field (1981).

### 2.3 Micro‐Computed Tomography

The functional morphology of the subgenual organ complex was analyzed by micro‐computed tomography (µCT) of a mesothoracic leg (midleg) from an adult female. The leg was cut off at the femur, fixed in Bouin's solution in 70% ethanol for 24 h, and washed and stored in 70% ethanol. It was stained in 0.3% phosphotungstic acid (PTA; Sigma‐Aldrich, St. Louis, MO, USA) in 70% ethanol (see Metscher 2009) for 12 days. Following the incubation, the leg was again washed in 70% ethanol. µCT‐scanning was done with a Skyscan 1272 (Bruker microCT, Kontich, Belgium) with the following parameters: tube voltage: 40 kV, tube current: 200 µA, sample rotation: 360°, step size: 1°, exposure time: 1000 ms, binning: 2 × 2, no filter, averaging: 4, random movement: 3, and voxel size: 1 µm.

Thermal drift correction and digital section reconstruction were performed in NRecon v2.2 (Bruker microCT). The resulting image stack was rotated in ImageJ (Abràmoff et al. 2004), where we also applied a CLAHE filter (Zuiderveld 1994), using a macro by Stefan Fischer (Fischer 2019). 2D images were analyzed in DataViewer 1.5 (Bruker microCT), and for volume renderings, we used Drishti v.3.0 (RRID:SCR_017999) (Limaye 2012).

### 2.4 Microscopy

Following tracing procedures, the leg preparations were viewed in methyl salicylate on a microscopy slide with an Olympus BH‐2 microscope (Olympus, Shinjuku, Japan). Digital images were acquired with a Leica DFC 7000 T camera (1920 × 1440 pixel), using the Leica Application Software LAS V4.9 (Leica Microsystems CMS GmbH, Wetzlar, Germany). Stacked photographs were generated from series of photographs with the freeware program CombineZP (combinezp.software.informer.com) (RRID:SCR_014567). The tibial nerve pattern was documented from a microscope (Leitz Wetzlar, Germany) with a drawing attachment (Leitz).

### 2.5 Documentation

The hand drawing of the tibial nerve pattern was digitally redrawn (CorelDraw 11; Corel, Ottawa, Canada; RRID:SCR_014235). Figure panels with microphotographs and individual µCT slices were assembled and labeled using CorelDraw 11. The length of the distal organ was measured in ImageJ (RRID:SCR_003070) (Abràmoff et al. 2004).

### 2.6 Statistics

Statistical analysis of sensillum numbers and length of the distal organ used Prism 4 (GraphPad, San Diego, CA, USA) (RRID:SCR_002798). All data were initially tested for normal distribution using the D'Agostino and Pearson omnibus normality test. Comparing the sensillum numbers for sensory organs as well as the distal organ lengths from different leg pairs used one‐way analysis of variance (ANOVA). For the nerve pattern, comparisons of proportions were carried out using the two‐sided Fisher's exact test.

## 3 Results

### 3.1 Neuroanatomy of the Subgenual Organ Complex

The subgenual organ complex in *B. rossius* consists of the subgenual organ and distal organ (Figure 1b). The subgenual organ spans the tibia in dorso‐ventral direction, and the distal organ extends in the distal direction of the tibia. A dense group of sensilla in the subgenual organ is termed the anterior‐ventral subgenual organ (avSGO) (Figures 1 and 3b). The dendrites and accessory cells of the subgenual and distal organs orient in distal direction of the tibia (subgenual organ: Figures 3b and 5a,b; distal organ: Figures 3f and 5a). The overall organization and nerve pattern of the subgenual organ complex are identical in all leg pairs (Figure 1b; Figure S1). Further, the numbers of sensilla in the sensory organs and the length of the distal organ are similar between leg pairs (Table 2). The subgenual organ contained 39–47 sensilla, and the differences in numbers of sensilla between leg pairs were statistically not significant (one‐way ANOVA: *p* = 0.4312, *F* = 0.8715, *R* ^2^ = 0.06771, df = 26; D'Agostino and Pearson omnibus normality test for individual leg pairs: *p* = 0.0745–0.9171). The distal organ contained 25–31 sensilla, and the differences in numbers of sensilla between leg pairs were statistically not significant (one‐way ANOVA: *p* = 0.7863, *F* = 0.2428, *R* ^2^ = 0.01983, df = 26; D'Agostino and Pearson omnibus normality test for individual leg pairs: *p* = 0.3472–0.6145).

**TABLE 2:** Number of sensilla in the sensory organs and length of the distal organ in *Bacillus rossius*. Abbreviations: DO, distal organ; SGO, subgenual organ.

	Prothoracic leg (*n* = 9)	Mesothoracic leg (*n* = 9)	Metathoracic leg (*n* = 9)
**SGO sensilla**	43 ± 4	45 ± 2	44 ± 3
**DO sensilla**	27 ± 1	27 ± 2	27 ± 2
**DO length ± *SD* (µm)**	519.30 ± 47.57	523.10 ± 55.19	558.00 ± 38.45

The length of the distal organ ranged between 452.4 µm (mesothoracic leg) and 626.2 µm (metathoracic leg). The differences in organ length between the thoracic leg pairs were statistically not significant (one‐way ANOVA: *p* = 0.1863, *F* = 1.904, *R* ^2^ = 0.1307, df = 26; D'Agostino and Pearson omnibus normality test for individual leg pairs: *p* = 0.1186–0.5220).

**FIGURE 2:**
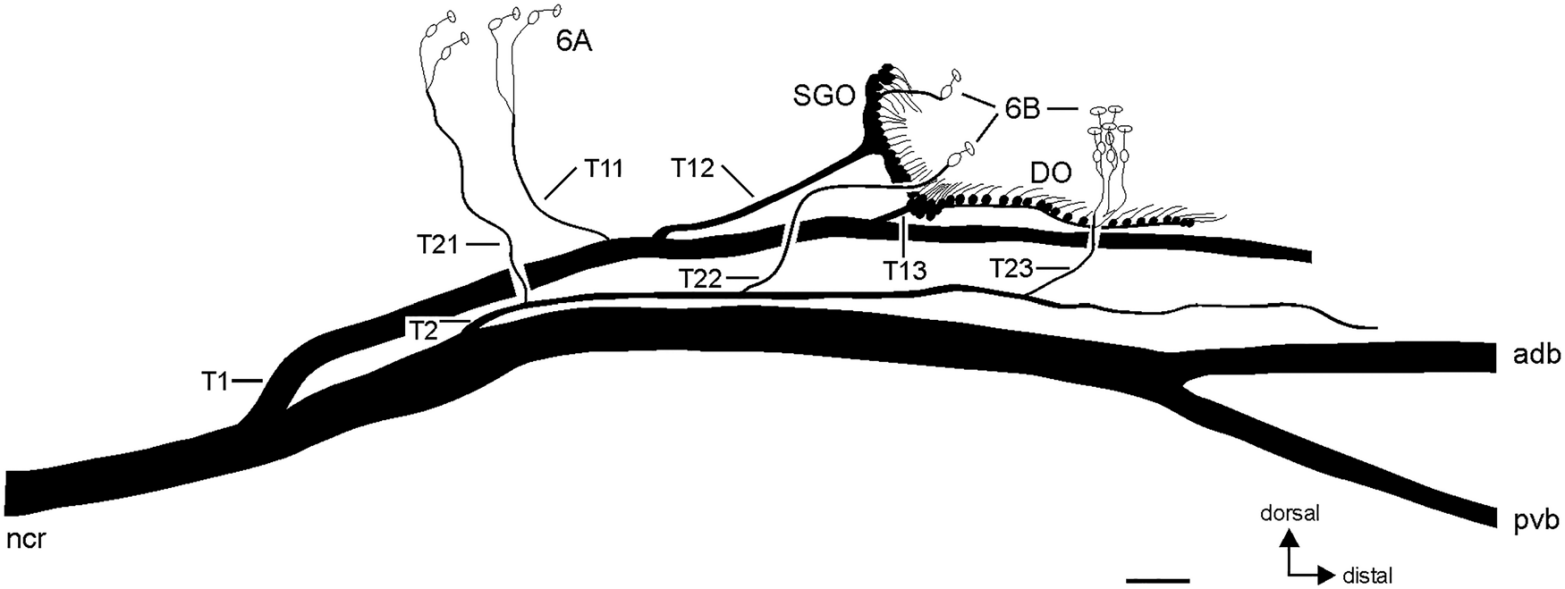
Schematic of the subgenual organ complex and campaniform sensilla in *Bacillus rossius* (mesothoracic leg), viewed from posterior side. For scolopidial sensilla, the cell bodies of sensory neurons are shown in black; for campaniform sensilla, the cell bodies of sensory neurons are shown in white. Tibial nerve branches from the nervus cruris are numbered successively from proximal to distal (T1, T12, T22, etc.). Scale = 100 µm. 6A/6B, groups of tibial campaniform sensilla; adb, anterior‐dorsal branch of nervus cruris; DO, distal organ; ncr; nervus cruris; pvb, posterior‐ventral branch of nervus cruris; SGO, subgenual organ.

**FIGURE 3:**
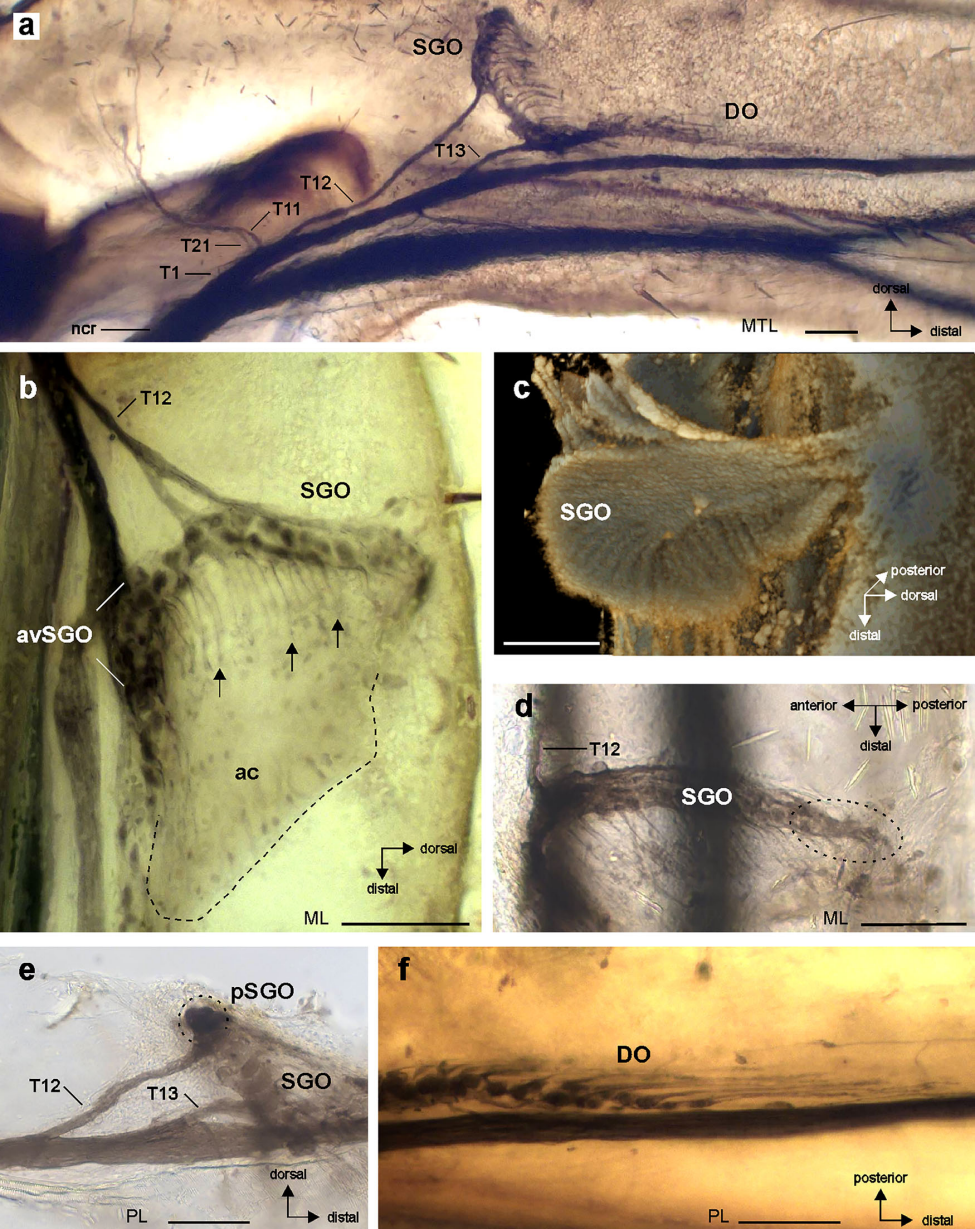
Neuroanatomy of the subgenual organ complex in *Bacillus rossius*. (a) Tracing preparation of the subgenual organ complex with staining of the subgenual organ (SGO) and the distal organ (DO) and some nerve branches and sub‐branches from nervus cruris identified (T1, T11, T12, T13, T21); see also Figure 2 for a schematic of the nerve pattern. Viewed from anterior. (b) The SGO with dendrites in distal direction (arrows) extends into accessory cells distally to the SGO (outlined by hatched line). The SGO is associated with nerve branch T12. Viewed from anterior. (c) Micro‐computed tomographic reconstruction of the SGO tissue, viewed from anterior‐ventral. (d) The posterior sensilla of the SGO viewed in dorsal perspective. The posterior sensilla (encircled with dotted line) have no separate nerve branch at the posterior side of the tibia; note nerve branch T12 at the anterior side of the SGO. (e) The posterior sensilla of the SGO (encircled) are continuous with the other subgenual sensilla, and associated with nerve branch T12. Viewed from posterior. (f) The most distal sensilla in the DO, note dendrites pointing in distal direction of the tibia. Viewed from anterior. Scales = 100 µm. avSGO, anterior‐ventral subgenual organ; DO, distal organ; ML, mesothoracic leg; MTL, metathoracic leg; PL, prothoracic leg; pSGO, posterior sensilla of the subgenual organ; SGO, subgenual organ.

The sensory elements of the tibia are associated with several branches of the nervus cruris (Figures 2 and 3a). The subgenual organ complex is associated with the first nerve branch in the tibia (T1) by two minor branches for the subgenual organ (T12, T13) and the distal organ (T13). The tibial nerve branch T1 is thus the only nerve branch associated with the chordotonal organs in the subgenual organ complex, and it splits from the nervus cruris at the femur‐tibia joint relatively close to the subgenual organ complex (Figure 3a, Figures S2 and S3). The subgenual organ is associated with nerve branch T12, and the sensilla at the posterior side of the subgenual organ are not associated with an additional, separate nerve branch (Figures 2a and 3d,e). Other nerve branches (T11, T21–T23) supply campaniform sensilla (Figure 2a) and hair sensilla in the proximal tibia. T1, T2, and nervus cruris extend in distal direction to the subgenual organ complex, the latter splitting into two branches (Figures 2 and 3a).

### 3.2 Variation of the Nerve Pattern

**FIGURE 4:**
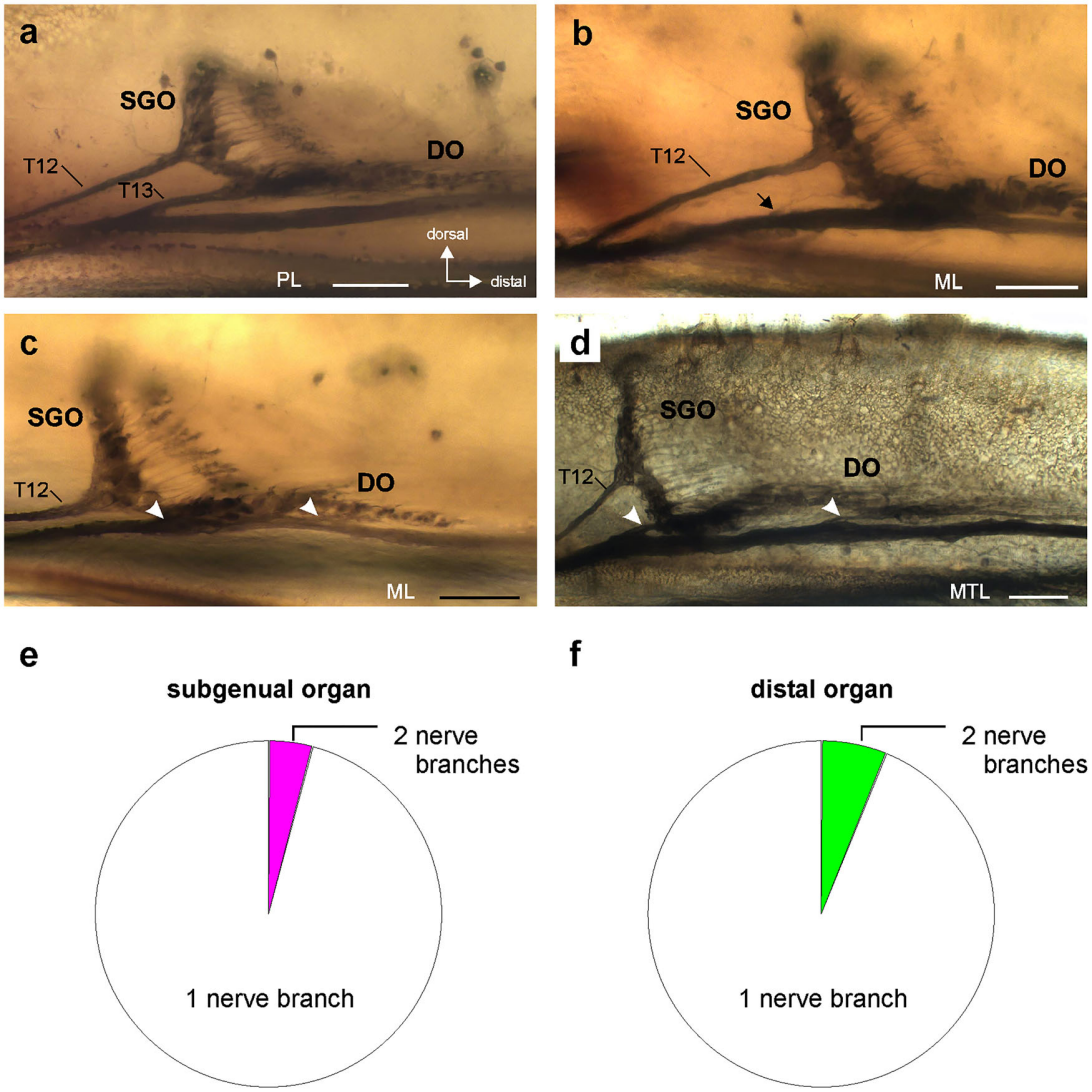
Variation of the nerve pattern in the sensory organs. (a) Axonal tracing preparation with a single nerve branch from the subgenual organ (SGO) (T12) and the distal organ (DO) (T13). (b) Preparation with two nerve branches from the SGO (arrow). (c and d) Preparations with two nerve branches from the DO (arrowheads). (a–d) viewed from anterior. (e) Proportions of leg preparations with one nerve (white) or two nerves (magenta) from the subgenual organ. (f) Proportions of leg preparations with one nerve (white) or two nerves (green) from the distal organ. Scales (a–d) = 100 µm. DO, distal organ; ML, mesothoracic leg; MTL, metathoracic leg; PL, prothoracic leg; SGO, subgenual organ.

The nerve pattern of sensory organs was highly consistent between different individuals and leg pairs. There is usually one nerve branch associated with the subgenual organ (T12; Figures 2, 3a,d,e, and 4a) and one nerve branch with the distal organ (T13; Figures 2, 3, and 4a). Only few cases were documented with differences in the T1 nerve branches for one of the sensory organs: In two preparations, the subgenual organ had two nerve branches to T1 (Figure 4b; both mesothoracic legs; 4.08% from 49 legs). The distal organ had a second branch in three preparations (Figure 4c,d; two mesothoracic legs, one metathoracic leg; 6.12% from 49 legs). The variation was thus rather limited for both organs, and the proportions of variations were similar between the subgenual organ and distal organ (Figure 4e,f) (two‐sided Fisher's exact test: *p* = 0.7475).

### 3.3 Functional Morphology of the Subgenual Organ Complex

**FIGURE 5:**
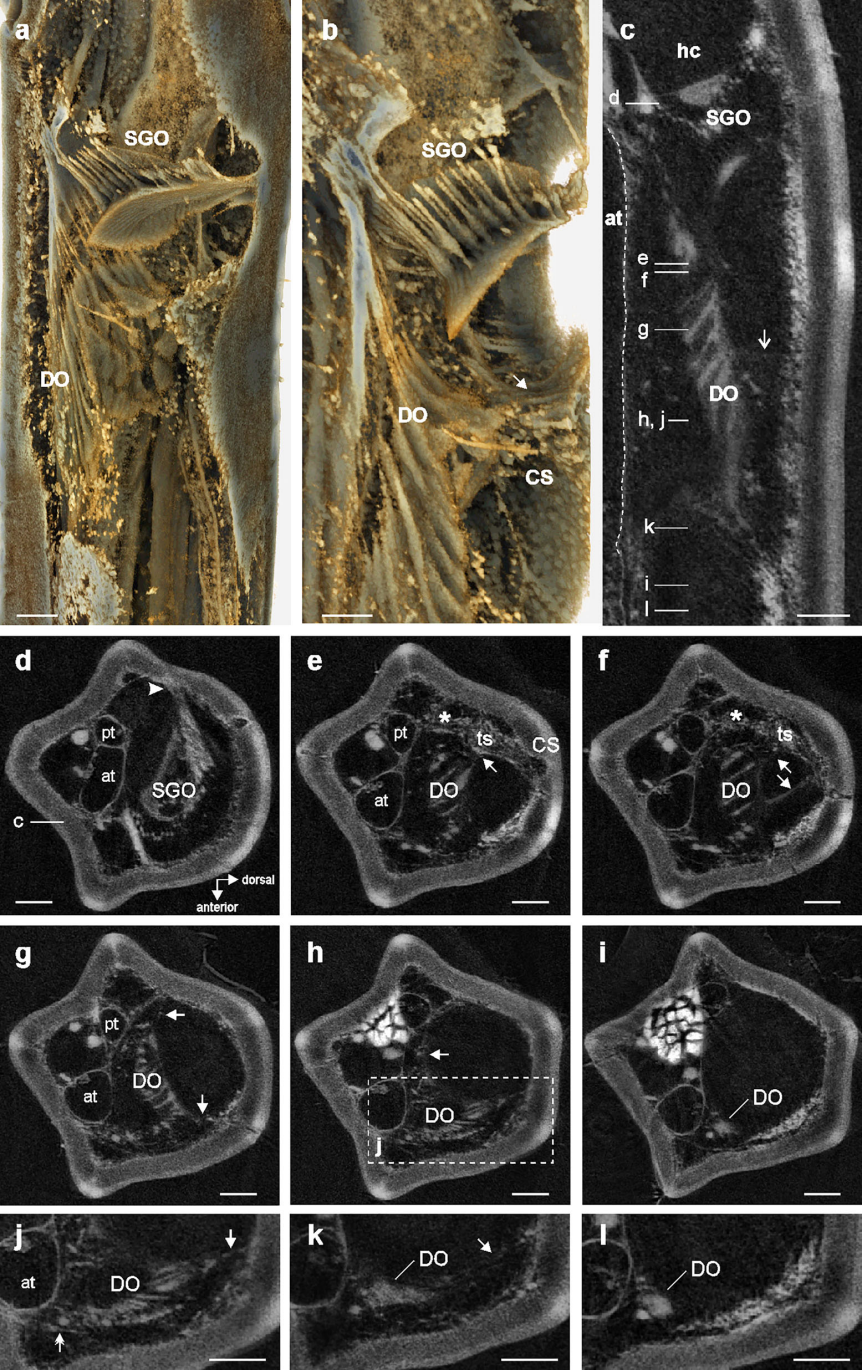
Functional morphology of the subgenual organ complex. (a and b) Reconstructions of the subgenual organ complex from µCT scan of the mesothoracic leg, (a) viewed from dorso‐anterior, (b) viewed from anterior. (c) Subgenual organ (SGO) and distal organ (DO) are placed in the hemolymph channel (hc) of the tibia. The central DO is closer to the dorsal cuticle, linked with some soft tissue (open arrow) (vertical longitudinal sections). The anterior trachea (at) is outlined with a hatched line. (d–i) Position and connection of the SGO and DO; attachment structures of the DO to the inner cuticle are indicated by arrows (transversal sections). Attachment of the SGO at the posterior tibia is indicated by the arrowhead in (d). Asterisk indicates the connective tissue to the posterior trachea (pt). (j–l) Details of the DO in the distal sections (transversal sections). Connection of the DO to the anterior trachea is indicated by the double‐headed arrow in (j). Scales = 100 µm. at, anterior trachea; CS, campaniform sensilla; DO, distal organ; hc, hemolymph channel; pt, posterior trachea; SGO, subgenual organ; ts, tissue strand.

The functional morphology concerns the structural organization, orientation, and connections of the sensory organs. Both sensory organs are placed in the hemolymph channel, located dorsally above the two tibial tracheae (Figure 5c,e–i). The subgenual organ is slightly tilted proximally at the dorsal tibia (Figures 3a–c and 5a–c). The subgenual sensilla terminate in a tissue containing the accessory cells (Figures 3b and 5a,b). This tissue spans the hemolymph channel and has a lamellate surface (Figures 3c and 5a). The tissue is slightly tilted at the dorsal tibia in proximal direction (Fig. 5‐ac). The subgenual organ has a strong attachment at the posterior tibia to the inner cuticle (Figure 5d). The accessory cells of the distal organ lay in parallel close to each other, resulting in a compact organ structure (Figure 5a–c,g,h). The distal organ is linked at the proximal end to the dorsal cuticle by a tissue strand at the level of the dorsal 6B campaniform sensilla (Figure 5b,e,f). In addition, from the dorsal side of the distal organ, a tissue strand connects towards the dorsal cuticle (Figure 5f,g). Over the length of the distal organ, thin strands of tissue occur at the posterior side to the posterior leg cuticle and at the anterior side to the dorsal cuticle (Figure 5g,h). These strands are not continuous with the dorsal strands at the proximal end (Figure 5f). At the distal end, the distal organ is linked to the dorsal cuticle by a fine tissue strand (Figure 5k). The distal tip is placed in the hemolymph and has no obvious attachment structures to other structure leg elements (Figure 5i,l). Hence, different attachment elements occur over the length of the distal organ to the cuticle, but the distal organ is placed in the hemolymph channel and the sensilla are not directly attached at the inner surface of the leg cuticle.

The distal organ was located dorsally to the two tracheae in the tibia (Figure 5e–i). At the proximal end of the distal organ, there is a gap towards the tracheae (Figure 5e). At this level, the tissue strand between the distal organ and the cuticle is also linked to the posterior trachea (the smaller trachea) (Figure 5e,f). This provides only an indirect link of the distal organ to the trachea. In the central region of the distal organ, the organ is connected to the anterior trachea at its dorsal side (Figure 5h,j). Further in distal direction, the distal organ is shifted in dorsal direction, whereas a tissue connection to the anterior trachea continues. Some strands also exist from the distal organ to the ventral cuticle (Figure S4). At its distal region, the distal organ is located dorsally to the anterior trachea, without showing a clear connection to the trachea (Figure 5i,l). Therefore, a connection to the trachea occurs in the proximal and central parts of the distal organ, but mostly indirectly by connective tissue rather than direct contact.

**FIGURE 6:**
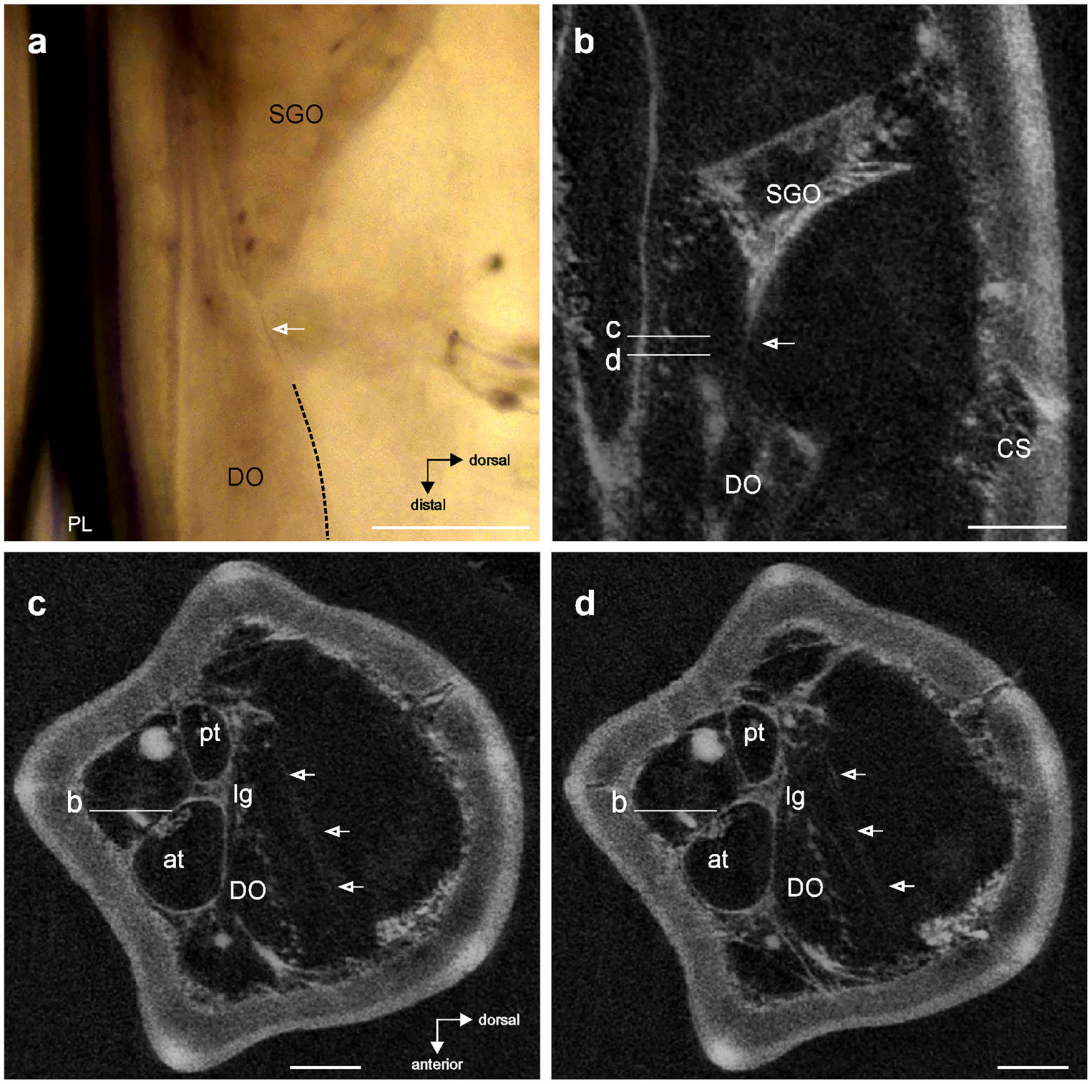
Morphological coupling of chordotonal organs in the subgenual organ complex. Levels of sections between (b)‐ (d) are indicated by labelled lines. (a) Membrane between the subgenual organ (SGO) and distal organ (DO) (indicated by empty arrow) by light microscopy (lateral view). The dorsal surface of the DO is indicated by hatched line. (b) µCT image shows the SGO is extended in distal direction and the sensory organs are connected by the membrane (arrow) (vertical longitudinal section). (c and d) The membrane extends from the anterior side towards the posterior side of the tibia (indicated by arrows) (transversal sections). Scales = 100 µm. at, anterior trachea; CS, campaniform sensilla; DO, distal organ; lg, ligament between the anterior and posterior trachea; PL, prothoracic leg; pt, posterior trachea; SGO, subgenual organ.

The subgenual organ tissue extends in distal direction (Figures 3b and 6a) and continues in a connection to the distal organ by a fine membrane (Figure 6a,b). Transversal sections show that this membrane is placed between the organs in the horizontal longitudinal plane between the anterior and posterior sides (Figure 6c,d).

## 4 Discussion

### 4.1 Neuroanatomy of the Subgenual Organ Complex in *B. rossius* and Comparison to Oyen (1901)

#### 4.1.1 Sensory Organs in the Subgenual Organ Complex

The subgenual organ complex in *B. rossius* consists of the subgenual organ and distal organ, consistent with sensory organs identified on the basis of homologies in Phasmatodea and other orthopteroid insects (Friedrich 1929). The overall neuroanatomical organization in *B. rossius* resembles the sensory organs documented for other stick insects such as *C. morosus* (Lonchodidae: Lonchodinae), *S. chlorotica* (Lonchodidae: Necrosciinae), *Ramulus artemis* Westwood, 1859 (Phasmatidae: Clitumninae), or *Oreophoetes peruana* Saussure, 1868 (Diapheromeridae: Diapheromerinae) (Strauß 2023a; Strauß and Lakes‐Harlan 2013; Strauß, Moritz, et al. 2021). This includes the avSGO group (Figures 1 and 3b) that was not described earlier in *B. rossius* (Oyen 1901). The analysis of sensory organs and related nerve branches by axonal tracing allows a detailed comparison to the earlier histological investigation of Oyen (1901).

#### 4.1.2 Nerve Pattern of the Subgenual Organ Complex

The nerve pattern found in *B. rossius* shows homologous nerve branches to other stick insects. There are two nerve branches associated with the chordotonal organs of the subgenual organ complex (T12 and T13), as described by Oyen (1901). The sensory nerve branch T1 originates at the femur‐tibia joint or at the proximal tibia. In no preparation was it found to occur more proximally in the middle of the femur (as stated by Oyen 1901). This origin more distally in the leg is consistent with the nerve branching in all other Phasmatodea studied so far.

#### 4.1.3 Nerve Pattern of the Posterior Subgenual Organ

The nerve pattern of the subgenual organ with one (T12) or two nerve branches (T12, T22) may provide a taxonomically relevant trait, as the subgenual organ in several Old World stick insects belonging to the Lonchodinae, Necrosciinae, and Clitumninae is associated with an anterior nerve branch (T12) and a posterior nerve branch (T22), including *C. morosus* (Friedrich 1929; Strauß and Lakes‐Harlan 2013). Oyen (1901) reported only one nerve branch related to the subgenual organ in *B. rossius*, and it was later assumed by Friedrich (1929) as present but not identified, based on considerations of homology from another stick insect species, *C. morosus*. However, the subgenual organ complex was recently shown to be associated with only T12 in New World stick insects (Occidophasmata; Strauß 2023a; 2023b) and the heteropterygid *Trachyaretaon echinatus* (Stål, 1877) (Oriophasmata: Heteropterygidae: Obriminae; Strauß 2025). Here, we confirm that in *B. rossius* as another Old World stick insect (Oriophasmata), the posterior sensilla of the subgenual organ are not associated with T22, as this nerve branch only supplies the posterior 6B campaniform sensillum (Figures 2 and 3d,e). This simpler nerve pattern for the subgenual organ with a single nerve branch is thus also present in certain clades of Oriophasmata. This distribution of the subgenual organ nerve pattern with a single nerve branch invites broader investigations in further groups of Oriophasmata, such as Lanceocercata, Cladomorphinae, or Phylliinae, to test whether the nerve pattern with two nerve branches is actually more restricted to certain groups within this clade than previously suggested and could represent the derived nerve pattern from a simpler pattern with a single nerve branch (Strauß 2025).

#### 4.1.4 Neuroanatomy of the Distal Organ

The size of the distal organ was depicted as notably short in the previous study and not even identified as a distinct sensory organ (Oyen 1901: subgenual organ II). Axonal tracing shows it to be longer in distal extension than the subgenual organ in all legs (Figures 1b and 3a), with linear sensilla as seen in other Phasmatodea. The numbers of sensilla in *B. rossius* are slightly higher than in *C. morosus*, *R. artemis*, or *S. chlorotica* (∼20; Strauß 2023a; Strauß and Lakes‐Harlan 2013). The numbers of sensilla in the distal organ may be species‐specific in Phasmatodea. This could be analyzed with additional species considering also body size, tibia length, and phylogenetic relatedness.

In sum, the subgenual organ complex in *B. rossius* resembles the neuroanatomy also documented in other Phasmatodea. These comparative findings also suggest a conserved mechanosensory function in the stick insects.

### 4.2 Variation of the Nerve Pattern

The nerve pattern for sensory organs in the subgenual organ complex is highly consistent in *B. rossius*: in most preparations, each sensory organ has a single nerve branch associated with T1. Notably, a higher degree in variation occurs for the distal organ than for the subgenual organ. Such neuroanatomical variation was also described for other stick insect species, but especially for the subgenual organ, the variation was more extensive (Strauß 2020, 2023a, 2023b). A variation with one or two nerve branches for each sensory organ likely originates during axonal pathfinding in embryonic development (see Strauß 2020 for discussion). The findings in *B. rossius* indicate a tight regulation by developmental mechanisms in the leg anlagen during embryogenesis. The molecular basis of these is so far not only analyzed in stick insects but likely also involves attractant and repellent molecular mechanisms of neurite orientation as in Orthoptera (Tessier‐Lavigne and Goodman 1996; Bonner et al. 2003).

### 4.3 Functional Morphology of the Distal Organ in *B. rossius*

#### 4.3.1 Attachment of the Distal Organ to the Leg Cuticle

For mechanosensory organs, the functional morphology by a specific mechanical coupling to transfer mechanical stimuli to the sensilla determines their physiological function (French 1988; Strauß et al. 2024). The distal organ extends along the main axis of the tibia and notably shows different attachments to the leg cuticle, whereas it is not closely fixed to it. At the proximal end, the largest tissue strand runs to the dorsale cuticle (Figure 5a,e). This strand also includes the axons of the dorsal 6B campaniform sensilla (Strauß 2022). The more distal parts showed strands to the dorsal and posterior cuticle (Figure 5f,g). At the more distal parts, the strands to the cuticle are finer, and the most distal section is not linked to the cuticle (Figure 5i,l). These strands are also found in other stick insects (Strauß, Moritz, et al. 2021). Notably, the distal organ is not strongly attached to the cuticle but placed in the hemolymph, similar to the subgenual organ. With respect to the mechanosensory function, the strands could position the distal organ within the hemolymph channel or provide a functional coupling to the transfer vibrations from the cuticle to the sensilla of the distal organ (Strauß and Stritih‐Peljhan 2022). The strands are rather thin, and the main exposition to mechanical stimuli for the distal organ is likely from the hemolymph.

#### 4.3.2 Connection of the Distal Organ to the Trachea

Further, a possible connection to the leg trachea is functionally relevant as a potential channel for sound input (Tettigonioidea: Schumacher 1979; Michelsen et al. 1994; Celiker et al. 2020) or for a transfer and amplifying function in vibration detection (Shaw 1994b). In *B. rossius*, the subgenual organ and distal organ are located dorsally to the tracheal branches within the tibia. The distal organ is linked by some connective tissue to the posterior trachea in the anterior and middle regions of the organ (Figure 5e,f) and is partly in contact to the anterior trachea at the dorsal side of the trachea in the middle region (Figure 5h,k). Its distal end is placed over the anterior trachea (Figure 5i,l). Hence, the distal organ is not closely coupled to the tracheae over its length. This finding is closer to the description on *C. morosus* where only part of the organ is in contact to the trachea (Friedrich 1929) than Oyen's (1901) description that the tracheae provide the structural basis for the chordotonal sensilla. In *R. artemis*, in the central region of the distal organ, a tissue strand connects the organ to the anterior trachea (Strauß, Moritz, et al. 2021), whereas in *B. rossi*, the contact area of the distal organ to the dorsal side of the anterior trachea is broader (Figure 5h). This connection to the trachea may also be relevant in the amplification of substrate vibrations (Shaw 1994b). However, the sensilla are not tightly linked to an expanded trachea present in auditory systems in Ensifera (Sickmann et al. 1997; Montealegre‐Z and Robert 2015; Hummel et al. 2017; Hall and Robinson 2021), and there is less direct contact to the trachea than in the cockroach distal organ (Schnorbus 1971). The tracheal branches in *B. rossius* show no further specializations such as enlarged tracheal vesicles that could relate to mechanosensory functions by providing a compressible space at the chordotonal organs that could amplify substrate vibrations (Shaw 1994b; Strauß 2021).

The subgenual organ contains the dendrites of the sensilla that run in parallel in distal direction (Figures 5a and 6b). It is less homogenous (Figure 6b) than seen in cockroaches (Schnorbus 1971; Moran and Rowley 1975). The organ has the strongest connection to the leg cuticle at the posterior tibia, and this is similar in other stick insects (Jägers‐Röhr 1968), cockroaches (Schnorbus 1971; Shaw 1994b), and Orthoptera (Friedrich 1929; Rössler 1992; Lin et al. 1994, 1995). The subgenual organ spans the hemolymph channel almost transversally and is therefore exposed to hemolymph movements. The connection between the two sensory organs by a membrane appears to be unique to stick insects and is suggested to provide a coupling that is functionally relevant in the response to mechanical stimuli (Strauß 2022). So far, the biomechanics of these sensory organs in stick insects are not analyzed, which depend on the position in the leg and the transfer of vibrations via the hemolymph and cuticle, the properties of attachments, and the accessory cells of sensilla (e.g., Vincent 2012; Prahlad et al. 2017; Strauß and Stritih‐Peljhan 2022). In particular, the connections by the membrane between sensory organs and the strands to the cuticle are expected to determine the displacements of the distal organ and the potential vibrational inputs.

## 5 Conclusion

This study documents the neuroanatomy and functional morphology of the subgenual organ complex in the stick insect *B. rossius*. The findings for the overall anatomy of sensory organs are consistent with the neuroanatomy and functional morphology in other Phasmatodea. The elaborate neuroanatomy indicates an adaptive function for mechanoreception, and the distal organ is likely receiving substrate vibrations mainly transferred via the hemolymph and, as indicated by its functional morphology, possibly also from the anterior tibial trachea. The diversification of neuronal structures in Phasmatodea concerns the number of sensilla in the sensory organs and the nerve pattern of the subgenual organ. The nerve pattern of the subgenual organ may be further analyzed in the species from the Phylliidae, from the African/Malagasy clade, and from the species‐rich Lanceocercata (Oriophasmata) (Bradler and Buckley 2018; Boisseau et al. 2025). These data may be included in further phylogeny‐based comparisons. So far, relatively few species of Phasmatodea are investigated, and a broader taxon sampling will allow us to match the neuroanatomical traits with phylogenetic relationships and may also support affinities between different clades of stick and leaf insects.

## Author Contributions

Conceptualization of the study: Johannes Strauß. Formal analysis – experiments: Johannes Strauß, Peter T. Rühr. Data analysis: Johannes Strauß, Peter T. Rühr. Reconstruction: Peter T. Rühr. Figure assembly: Johannes Strauß. Writing manuscript (original draft): Johannes Strauß, Peter T. Rühr. Writing manuscript (final script): Johannes Strauß, Peter T. Rühr. Editing of the manuscript: Johannes Strauß, Peter T. Rühr. Funding acquisition: Johannes Strauß. Both authors read the manuscript and agreed with the publication.

## Funding

Funded by the Deutsche Forschungsgemeinschaft (DFG, German Research Foundation)—STR 1329/2‐1. The funding agency did not play a role in the design of the study, in the collection, analyses, or interpretation of data, or the writing of the manuscript, and the decision to submit the study for publication.

## Supporting information




**Supplementary Figure S1‐S4**: cne70126‐sup‐0001‐FigureS1‐S4.pdf

## Data Availability

The µCT scan used in this study can be accessed at Zenodo: doi.org/10.5281/zenodo.14168668. The data on the sensillum numbers in the sensory organs are available as Supporting Information section.
