# Supplementary material for: The Mechanosensory Subgenual Organ Complex in the Stick Insect Bacillus rossius (Phasmatodea): Neuroanatomy and Functional Morphology
Source: J Comp Neurol. 2026 Jan 10;534(1):e70126. doi: 10.1002/cne.70126 (PMC12789965; doi:10.1002/cne.70126)
Supplement: Supplementary file 1 — Supplementary Figure S1‐S4: cne70126‐sup‐0001‐FigureS1‐S4.pdf [file CNE-534-e70126-s001.pdf]

Supplementary Figures

Supplementary Fig. 1: Tracing preparations of the subgenual organ complex from all leg pairs. (a) prothoracic leg, (b) mesothoracic leg, (c) metathoracic leg.

Abbreviations: DO, distal organ; ML, mesothoracic leg; MTL, metathoracic leg; PL, prothoracic leg; SGO, subgenual organ.

Scales = 100 µm.

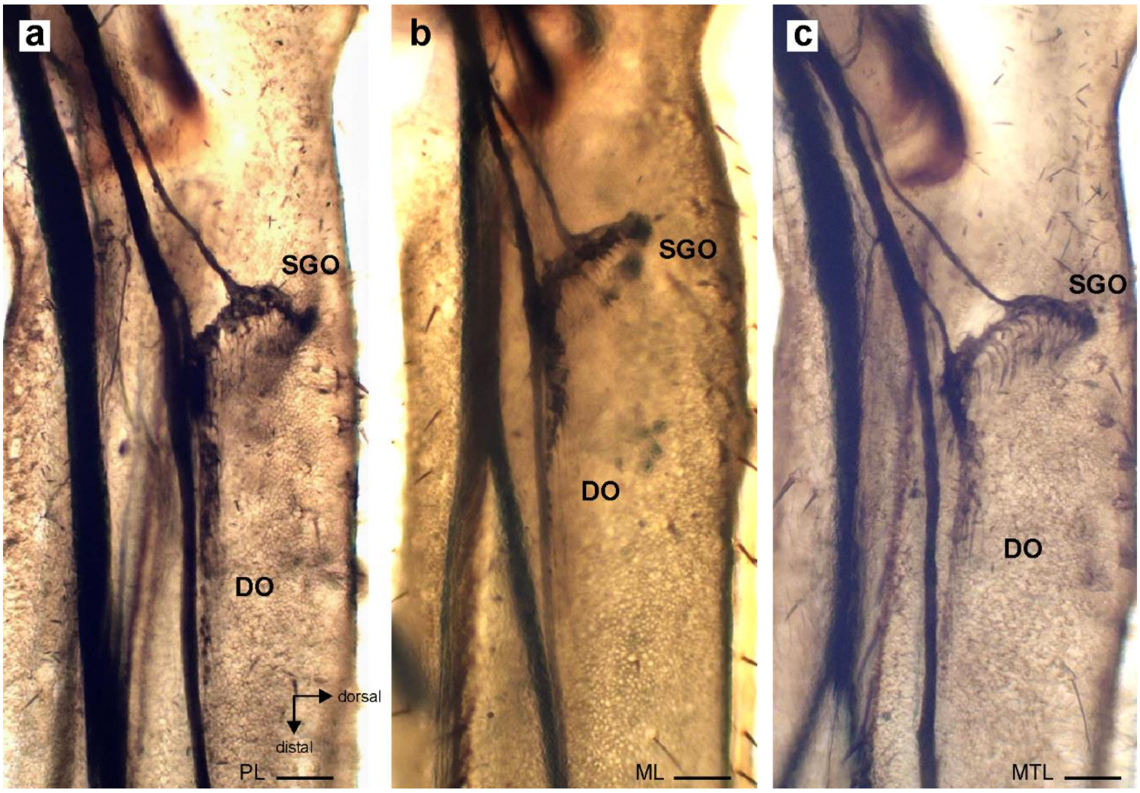

Supplementary Fig. 2: Origin of nerve branch T1 in the leg at the femur-tibia joint (in situ-staining in the mesothoracic leg).

Scale = 100 µm.

Abbreviations: DO, distal organ; ML, mesothoracic leg; ncr, nervus cruris; SGO, subgenual organ.

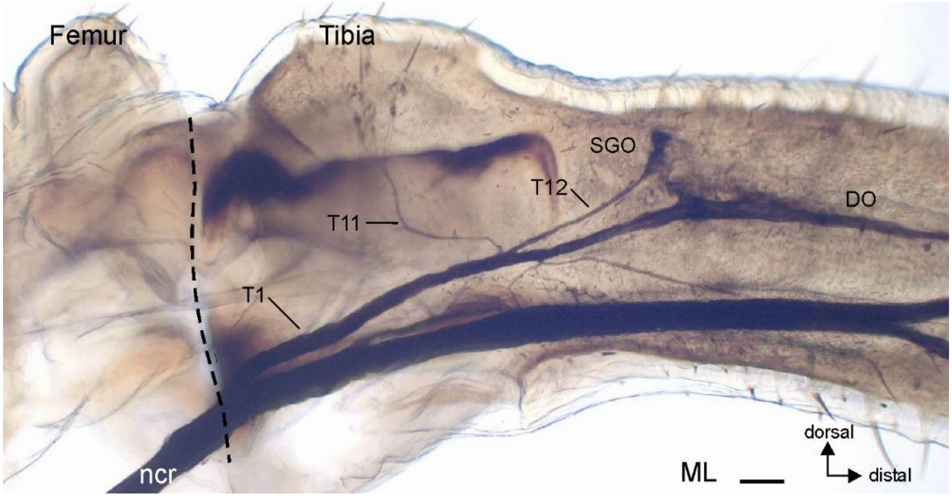

Supplementary Fig. 3: Ventral view of T1 as it splits off the nervus cruris (ncr). Preparation of a prothoracic leg.

Scale = 100 µm.

Abbreviations: DO, distal organ; PL, prothoracic leg; ncr, nervus cruris; SGO, subgenual organ.

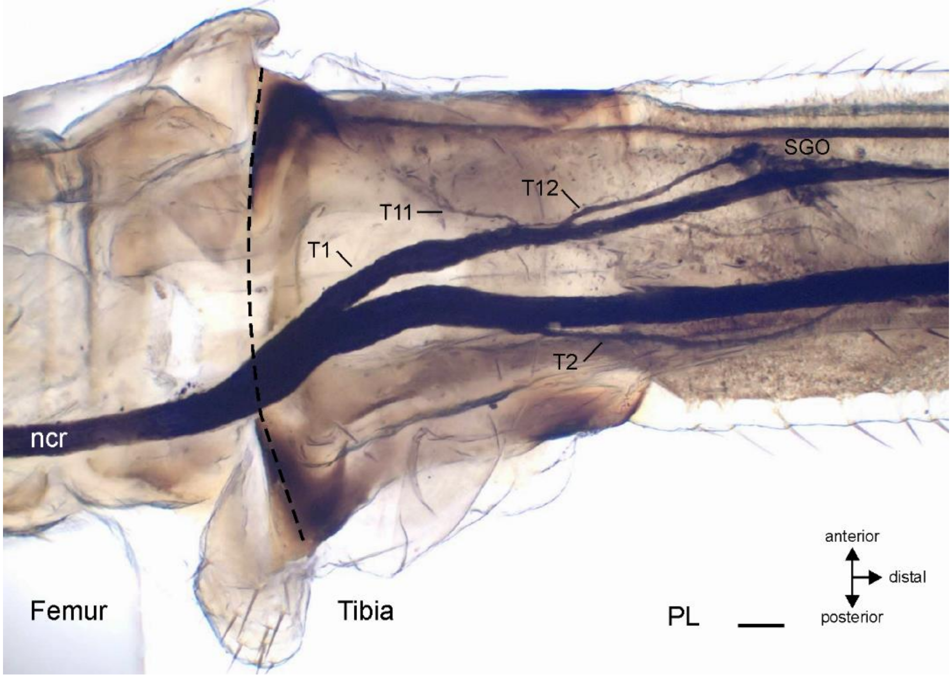

Supplementary Fig. 4: The functional morphology of the central part of the distal organ. The vertical longitudinal section of the tibia (see Fig. 5c) indicates the levels of sections in (a-c). (**a-c**) Transverse sections of the tibia with the distal organ (DO) located above the anterior trachea (outlined by the hatched line). The DO is raised to the dorsal cuticle and linked with a tissue strand (arrow). At the ventral side, a strand also runs to the cuticle (double-headed arrow).

Scales = 100 µm.

Abbreviations: at, anterior trachea; DO, distal organ; lg, ligament; pt, posterior trachea; SGO, subgenual organ.

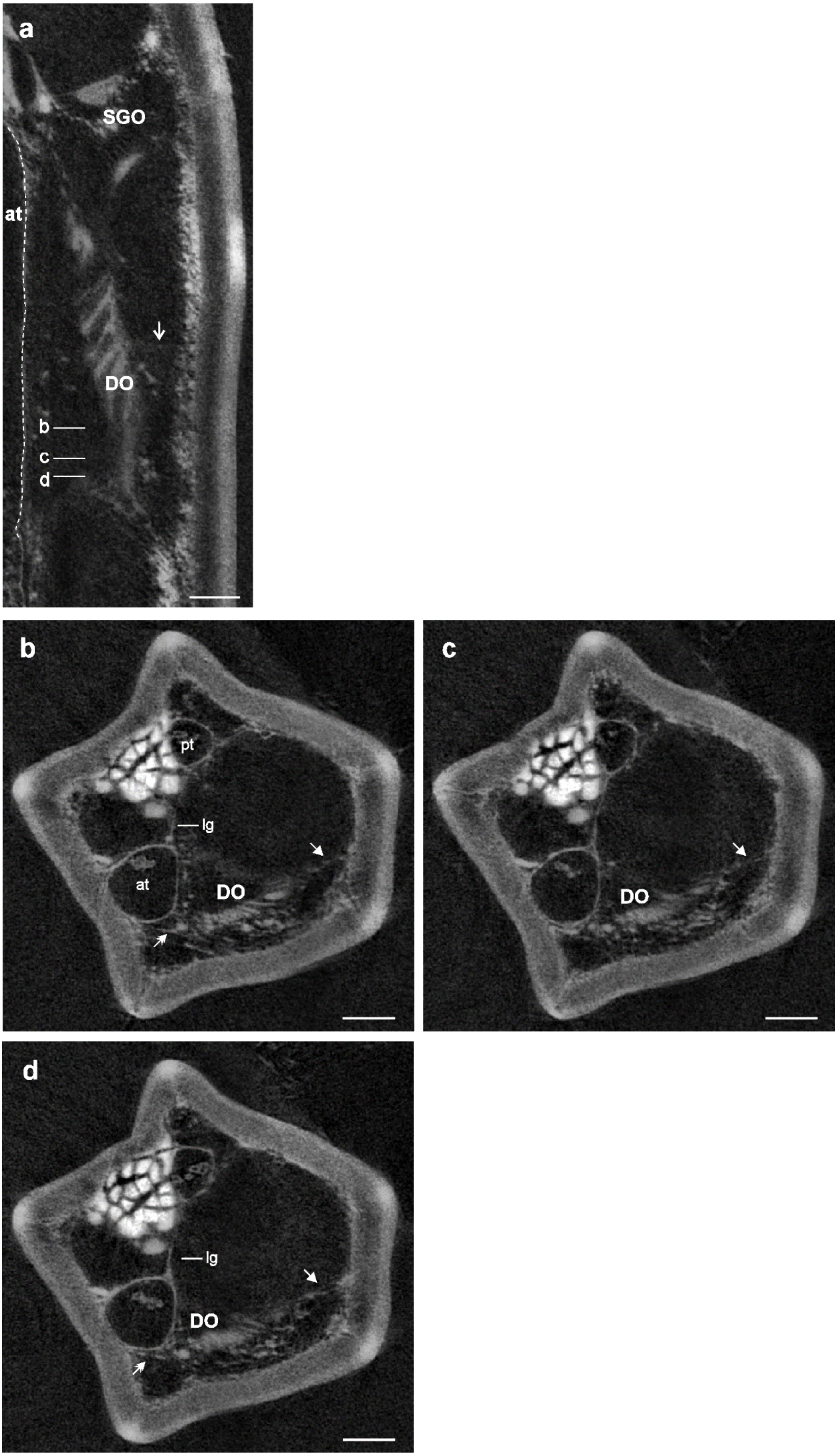
a
SGO
at
DO
b
c
d
pt
lg
at
DO
c
DO
d
lg
DO
